# From Neonatal History to Adult Cardiovascular Risk: Preterm Birth as a Lifelong Risk Enhancer

**DOI:** 10.3390/medsci14050543

**Published:** 2026-09-03

**Authors:** Mohamedanas Mohamedfaruk Patni, Malek Nasrallah, Dana Ja’afer Al Tarawneh, Mohamed El-Tanani, Khalid Ahmed Ibrahim, Abdullah Mohamad Hajjo, Mhd Munzer Zaher Hussin Alali, Ibrahim Alabid, Wasim Iyad Alghoul, Sakina Abdeali Sehrawala, Imran Rangraze, Ramya Kundayi Ravi

**Affiliations:** 1 Department of Community Medicine, RAK College of Medical Sciences, RAK Medical and Health Sciences University, Ras Al Khaimah 11172, United Arab Emirates; mohamedanas@rakmhsu.ac.ae; 2 Medical Internship Program, Kuwait Hospital, Emirates Health Services, Sharjah 32223, United Arab Emirates; dr.maleknas@gmail.com (M.N.); munzer.alali18@hotmail.com (M.M.Z.H.A.); 3 Medical Internship Program, Emirates Health Services, Ras Al Khaimah 11172, United Arab Emirates; dana.20901078@rakmhsu.ac.ae; 4 RAK College of Pharmacy, RAK Medical and Health Sciences University, Ras Al Khaimah 11172, United Arab Emirates; eltanani@rakmhsu.ac.ae; 5 Medical Internship Program, Al Qassimi Hospital, Emirates Health Services, Sharjah P.O. Box 3500, United Arab Emirates; khaliidibrahiim02@gmail.com; 6 Medical Internship Program, Umm Al Quwain Hospital, Emirates Health Services, Umm Al Quwain P.O. Box 24, United Arab Emirates; asqabood1243@gmail.com; 7 RAK College of Medical Sciences, RAK Medical and Health Sciences University, Ras Al Khaimah 11172, United Arab Emirates; wasim.21901111@rakmhsu.ac.ae; 8 Medical Internship Program, Sheikh Khalifa Medical City, Abu Dhabi 51900, United Arab Emirates; sakina.20901010@rakmhsu.ac.ae; 9 Department of Internal Medicine, RAK College of Medical Sciences, RAK Medical and Health Sciences University, Ras Al Khaimah 11172, United Arab Emirates; 10 RAK College of Nursing, RAK Medical and Health Sciences University, Ras Al Khaimah 11172, United Arab Emirates; ramya@rakmhsu.ac.ae

**Keywords:** preterm birth, prematurity, cardiovascular risk, birth history, preventive cardiology, hypertension, cardiac remodeling, pulmonary vascular disease, chronic kidney disease, cardiometabolic risk

## Abstract

Background: Preterm birth is increasingly recognized as an early-life exposure with potential cardiovascular consequences across the lifespan. Although improved neonatal care has increased survival among individuals born very and extremely preterm, birth history remains rarely incorporated into cardiovascular risk assessment. Objective: This review synthesizes current evidence linking preterm birth with cardiovascular risk and proposes a practical birth-history-informed framework for preventive cardiology. Methods: A narrative review was conducted to evaluate evidence from population-based cohort studies, sibling-comparison analyses, cardiovascular imaging studies, physiologic exercise studies, Mendelian randomization analyses, mechanistic studies, and selected reviews. The review focused on cardiovascular outcomes, blood pressure trajectories, cardiac remodeling, pulmonary vascular disease, renal and metabolic pathways, autonomic regulation, exercise physiology, and clinical prevention. Results: Preterm birth is most consistently associated with higher blood pressure across life stages and with altered cardiac structure or reserve, particularly among individuals born very or extremely preterm or with complicated neonatal courses. Population-based evidence also links preterm birth with later heart failure, ischemic heart disease, stroke, atrial fibrillation, chronic kidney disease, and diabetes, although absolute risks in young adulthood remain low and associations vary by gestational age, fetal growth, and neonatal complications. Pulmonary vascular, right-heart, and autonomic findings appear most relevant to selected high-risk or symptomatic survivors rather than to all individuals born preterm. Current evidence supports targeted prevention rather than universal advanced testing. Conclusions: Preterm birth should be viewed as an early-life cardiovascular risk enhancer rather than a forgotten neonatal detail. Incorporating gestational age, birth weight, and neonatal complications into cardiovascular history-taking may improve early risk recognition, guide blood pressure and cardiometabolic surveillance, and support individualized prevention across the lifespan.

## 1. Introduction

Cardiovascular prevention in adults is commonly guided by structured cardiovascular risk assessment, including prediction tools such as SCORE2 in European practice, together with clinical interpretation of modifiable and non-modifiable risk factors. These models emphasize adult variables such as age, sex, smoking status, blood pressure, lipids, diabetes, obesity, and family history, but they generally do not incorporate early-life exposures such as gestational age, birth weight, or neonatal complications [1,2]. However, a growing body of evidence suggests that cardiovascular vulnerability may begin much earlier, including during fetal and neonatal life. Preterm birth, defined as birth before 37 completed weeks of gestation, is increasingly recognized as a non-modifiable early-life exposure associated with long-term cardiovascular risk [3,4]. For clarity, standard neonatal definitions are used throughout this review: low birth weight (LBW) refers to birth weight below 2500 g, very low birth weight (VLBW) to birth weight below 1500 g, very preterm birth to birth before 32 completed weeks of gestation, and extremely preterm birth to birth before 28 completed weeks of gestation. As survival after very and extremely preterm birth continues to improve, clinicians are increasingly likely to encounter adolescents and adults whose cardiovascular risk profile may partly reflect developmental disruption rather than only acquired adult risk factors [5]. The cardiovascular relevance of prematurity is supported by large population-based studies linking preterm birth with major cardiovascular outcomes. Preterm birth has been associated with increased long-term risk of heart failure and ischemic heart disease in adulthood [6,7]. It has also been linked with higher risks of stroke and atrial fibrillation, suggesting that its consequences are not limited to ventricular dysfunction or hypertension alone [8,9]. These findings support the concept that prematurity may act as an early-life cardiovascular risk enhancer, particularly among individuals born very or extremely preterm, those with very low birth weight, or those exposed to neonatal complications [10,11].

Blood pressure is the most practical clinical entry point for recognizing this risk. Studies have reported higher blood pressure, altered blood pressure trajectories, and abnormal hemodynamic responses to exercise among individuals born preterm [12,13]. In childhood, prematurity combined with neonatal intensive care unit admission and neonatal complications has been associated with a higher risk of persistent hypertension, supporting the need for earlier surveillance in selected high-risk groups [11]. Because elevated blood pressure in childhood and adolescence can track into adulthood, systematic recognition of preterm birth may create an opportunity for prevention before overt cardiovascular disease develops.

Imaging studies have demonstrated altered left ventricular geometry and atrial remodeling in adolescents and adults born preterm [14,15]. In some individuals, smaller cardiac dimensions may coexist with preserved resting function, whereas abnormal cardiovascular responses may become more apparent during hypoxia [16,17]. This compensated phenotype is clinically important because conventional resting assessment may underestimate cardiovascular vulnerability in patients born preterm.

The mechanisms linking preterm birth to cardiovascular disease are likely multisystemic. Prematurity may interrupt cardiac, vascular, pulmonary, renal, autonomic, and metabolic development during critical periods of maturation [5,18]. Chronic kidney disease and diabetes have also been reported more frequently after preterm birth, providing additional pathways through which early-life developmental disruption may amplify later cardiovascular risk [19,20]. Therefore, prematurity should not be viewed as a single-organ exposure, but as a developmental condition with potential consequences across the cardiometabolic and cardiopulmonary spectrum.

Despite this evidence, birth history is not routinely incorporated into cardiovascular risk assessment. Most adult risk models do not include gestational age, birth weight, neonatal intensive care admission, bronchopulmonary dysplasia, neonatal hypertension, or neonatal kidney injury. This gap may lead clinicians to miss an important risk-enhancing exposure, particularly in young patients with early hypertension, unexplained exercise intolerance, premature heart failure, pulmonary vascular disease, or early cerebrovascular events. This review aims to synthesize evidence linking preterm birth with cardiovascular risk across the lifespan and to propose a practical birth-history-informed framework for cardiovascular assessment and prevention.

## 2. Materials and Methods

### 2.1. Study Design

This article was designed as a narrative review with a structured literature search. The objective was to synthesize clinical, epidemiological, imaging, physiologic, renal, metabolic, and mechanistic evidence linking preterm birth with cardiovascular risk across the lifespan and to translate these findings into a practical birth-history-informed cardiovascular risk-assessment framework.

### 2.2. Data Sources and Search Strategy

The literature search was conducted using PubMed/MEDLINE, Scopus, Google Scholar, and journal-specific databases. The search period covered studies published from January 2010 to July 2026. Older foundational studies were included when they were directly relevant to key mechanisms or clinical outcomes. Search terms were used alone and in combination, including: “preterm birth,” “prematurity,” “very low birth weight,” “gestational age,” “birth history,” “cardiovascular disease,” “preventive cardiology,” “hypertension,” “blood pressure,” “heart failure,” “ischemic heart disease,” “stroke,” “atrial fibrillation,” “cardiac remodeling,” “left ventricle,” “right ventricle,” “arterial stiffness,” “pulmonary vascular disease,” “bronchopulmonary dysplasia,” “chronic kidney disease,” “diabetes,” “autonomic dysfunction,” “exercise capacity,” and “cardiovascular risk assessment.”

### 2.3. Study Selection Process

Titles and abstracts were screened for relevance to preterm birth and cardiovascular, cardiometabolic, renal, pulmonary vascular, autonomic, or prevention-related outcomes. Full-text articles were assessed when the title or abstract suggested direct relevance to the review objective. Priority was given to population-based cohort studies, sibling-comparison analyses, longitudinal follow-up studies, cardiovascular imaging studies, physiologic exercise studies, Mendelian randomization analyses, mechanistic studies, and clinically relevant reviews.

The search and initial screening were performed by members of the author group. Potentially relevant full-text articles were reviewed by the author group for relevance to the review objective, clinical importance, methodological strength, and representation of the main evidence domains. Any uncertainty regarding inclusion was resolved through discussion and consensus among the authors. Because this was a narrative review, study selection was based on relevance to the review objective, clinical importance, methodological strength, and representation of the main evidence domains rather than formal systematic-review ranking.

### 2.4. Inclusion Criteria

Studies were eligible if they evaluated individuals born preterm or with very low birth weight and reported cardiovascular outcomes, blood pressure trajectories, cardiac structure or function, vascular physiology, pulmonary vascular or right-heart findings, chronic kidney disease, diabetes, autonomic regulation, exercise capacity, or prevention-related clinical implications. Human studies were prioritized. Reviews were included only when they provided relevant conceptual, mechanistic, or clinical context.

### 2.5. Exclusion Criteria

Non-peer-reviewed reports, conference abstracts without full-text data, studies without direct relevance to preterm birth or cardiovascular/cardiometabolic outcomes, and articles focused exclusively on unrelated neonatal or non-cardiovascular outcomes were excluded.

### 2.6. Data Synthesis and Statistical Analysis

Findings were synthesized narratively by clinical domain. No quantitative pooling or statistical analysis was performed because the included evidence was heterogeneous in design, population, age at assessment, exposure definition, outcomes, and measurement methods. The aim was not to estimate a pooled effect size, but to integrate available evidence into a clinically practical framework for cardiovascular risk assessment and prevention.

### 2.7. Limitations of the Selection Process

The selection process has limitations inherent to narrative reviews. Although a structured literature search was used, this review was not designed as a systematic review and was not prospectively registered. Therefore, PRISMA reporting, duplicate independent screening of all records, formal risk-of-bias scoring, and quantitative meta-analysis were not performed. To improve transparency, the databases searched, search terms, study period, inclusion and exclusion criteria, selection approach, and method of narrative synthesis are described above.

## 3. Why Preterm Birth Should Be Viewed as a Cardiovascular Risk Enhancer

A cardiovascular risk enhancer is a clinical factor that may not independently define disease, but modifies how baseline risk should be interpreted. Preterm birth fits this concept because it is a non-modifiable early-life exposure that occurs before conventional adult risk factors emerge and may influence cardiovascular structure, physiology, and reserve across the lifespan [3]. Unlike smoking, diet, obesity, or dyslipidemia, prematurity is not an acquired adult exposure. Rather, it represents a developmental event that may alter the biological foundation on which later cardiovascular risk accumulates [5].

The rationale for this framing is supported by both clinical outcomes and subclinical cardiovascular findings. Large population-based studies have associated preterm birth with increased risk of heart failure and ischemic heart disease in adulthood [6,7]. Additional cohort evidence links preterm birth with stroke and atrial fibrillation, suggesting that its cardiovascular relevance extends beyond hypertension or ventricular dysfunction alone [8,9]. However, this risk is unlikely to be uniform. Gestational age, fetal growth, birth weight, neonatal intensive care unit admission, bronchopulmonary dysplasia, neonatal hypertension, and other complications may all influence later vulnerability [10,11].

Preterm-associated cardiovascular risk may also differ from traditional adult cardiovascular disease in its biological origin. In the general population, cardiovascular disease often develops after years of cumulative exposure to hypertension, insulin resistance, obesity, dyslipidemia, inflammation, smoking, and physical inactivity. In contrast, prematurity may begin with interrupted cardiac, vascular, pulmonary, renal, autonomic, and metabolic maturation during a critical developmental window [5]. This distinction is important because preterm-born individuals may enter childhood and adulthood with measurable differences in organ structure or physiologic reserve before conventional risk scores identify them as high risk.

Cardiac imaging studies support this developmental phenotype. Adults and adolescents born preterm have been reported to show altered left ventricular geometry and atrial remodeling [14,15]. Some cohorts have also demonstrated smaller cardiac dimensions with preserved resting function, while abnormalities may become more apparent during physiologic stress such as hypoxia [16,17]. This compensated pattern has practical implications: a patient born preterm may appear clinically stable at rest while still having reduced cardiovascular reserve under increased hemodynamic demand.

Blood pressure is the most accessible clinical marker through which this risk can be recognized. Preterm birth has been associated with higher blood pressure in childhood, adolescence, and adulthood, as well as exaggerated blood pressure responses during exercise [13,21]. Recent evidence suggests that preterm infants requiring neonatal intensive care, particularly those with major neonatal complications, have a higher risk of persistent childhood hypertension [11]. Because elevated blood pressure early in life can track into adulthood, birth history may help identify individuals who would benefit from earlier surveillance and prevention.

The risk-enhancer model is further strengthened by renal, metabolic, pulmonary vascular, and autonomic pathways. Preterm birth has been associated with chronic kidney disease, smaller renal size, altered renin–angiotensin physiology, and higher ambulatory blood pressure, supporting a renal-developmental pathway to later cardiovascular risk [18,19]. It has also been linked with diabetes and altered autonomic regulation, both of which may amplify cardiometabolic vulnerability over time [20,22]. In selected survivors, pulmonary vascular disease and impaired right ventricular–vascular coupling may contribute to dyspnea, exercise limitation, and right-heart susceptibility [23,24].

Therefore, preterm birth should be viewed as an early-life cardiovascular risk enhancer rather than a stand-alone diagnosis. Its presence should not automatically lead to advanced testing in every individual, but it should prompt more complete birth-history assessment, earlier blood pressure and cardiometabolic screening, and greater attention to symptoms such as unexplained dyspnea, exercise intolerance, early-onset hypertension, or premature cardiovascular disease. This balanced approach recognizes prematurity as clinically meaningful without overmedicalizing all individuals born preterm.

## 4. Birth-History-Informed Cardiovascular Risk Assessment

### 4.1. Rationale for Incorporating Birth History

Birth history is a simple, low-cost, and clinically meaningful component of cardiovascular assessment. Unlike advanced imaging, genetic testing, or specialized biomarkers, it can be obtained through routine history-taking and may identify patients whose cardiovascular vulnerability began before the development of conventional adult risk factors. This is especially relevant in adolescents and young adults with early-onset hypertension or chronic kidney disease [11,19], diabetes [20], or premature major cardiovascular disease, including heart failure and ischemic heart disease [6,7], as well as stroke and atrial fibrillation [8,9]. Birth history may also provide important clinical context in patients with unexplained exercise intolerance, dyspnea, or abnormal right-heart findings [23,25,26].

The purpose of incorporating birth history is not to classify every individual born preterm as high risk. Rather, it allows clinicians to interpret current findings within a developmental context. Mild blood pressure elevation, reduced exercise tolerance, early metabolic disease, renal dysfunction, or unexplained pulmonary vascular findings may carry greater clinical significance in someone born very preterm or with a complicated neonatal course [11,23]. In this way, preterm birth functions as a risk-enhancing variable: it does not replace standard risk assessment, but it can refine clinical judgment when risk appears premature, borderline, or incompletely explained.

### 4.2. Practical Birth-History Questions

A birth-history-informed cardiovascular assessment should be brief, practical, and easy to incorporate into routine clinical history-taking. The goal is not to reconstruct the entire neonatal course, but to identify key early-life features that may modify cardiovascular interpretation in adolescence and adulthood. This is particularly relevant because neonatal intensive care admission, major neonatal complications, neonatal hypertension, and fetal growth restriction may identify individuals with greater blood-pressure vulnerability [10,11], while bronchopulmonary dysplasia may indicate greater pulmonary vascular or right-heart vulnerability [23]. Renal and metabolic risk should be interpreted separately using the evidence linking preterm birth with chronic kidney disease and diabetes [19,20]. The suggested minimum questions are summarized in Table 1.

These questions should be interpreted pragmatically. Many adults will not know their exact gestational age or birth weight, but a history of being born “very early,” requiring prolonged neonatal intensive care, or needing oxygen support can still be clinically informative. When available, neonatal discharge summaries, pediatric records, or parental history can help clarify the severity of prematurity and the presence of complications. The information obtained from Table 1 should then guide risk interpretation rather than automatically trigger advanced testing in every individual born preterm. Maternal pregnancy history should be interpreted as contextual rather than diagnostic, because pregnancy vascular disorders, especially preeclampsia, may partly explain the association between preterm delivery and later maternal cardiovascular disease and may provide useful background when interpreting pregnancy-related vascular risk [33].

### 4.3. Risk Interpretation in Clinical Practice

A graded approach is more appropriate than a universal testing strategy. Individuals born late preterm without known neonatal complications may only require routine cardiovascular prevention and standard blood pressure monitoring. In contrast, those born very or extremely preterm, those with very low birth weight, or those with bronchopulmonary dysplasia or neonatal hypertension may warrant closer clinical attention [29,31]. Fetal growth restriction and persistent childhood hypertension should also increase concern during risk interpretation [10,11]. Evidence of right-heart structural differences after extreme prematurity and right-ventricular dysfunction associated with bronchopulmonary dysplasia further supports closer attention in symptomatic or higher-risk individuals [30,34].

Risk should also be interpreted in combination with current clinical factors. A preterm-born patient with normal blood pressure, healthy weight, no cardiometabolic abnormalities, and no symptoms should not be overmedicalized. However, a similar birth history in a patient with early hypertension, reduced exercise capacity, abnormal renal function, diabetes, dyspnea, or premature cardiovascular disease should lower the threshold for more detailed evaluation. This approach is clinically balanced because it recognizes prematurity as relevant without treating it as deterministic.

### 4.4. Suggested Screening and Escalation Approach

For individuals with a known history of preterm birth, the minimum assessment should include accurate blood pressure measurement, body mass index, smoking and vaping history, physical activity level, diet, sleep, and family history of cardiovascular disease. Blood pressure assessment should follow contemporary hypertension guidance, including standardized office measurement and confirmation of elevated readings with repeat office or out-of-office measurements when appropriate [35]. This is particularly important in preterm-born individuals because small but persistent blood pressure differences may influence long-term cardiovascular risk interpretation, especially when combined with renal, metabolic, or cardiopulmonary vulnerability. Blood pressure deserves particular emphasis because it is practical, modifiable, and repeatedly linked with preterm birth across childhood, adolescence, and adulthood [13,21].

The timing of assessment should be pragmatic because no guideline currently defines fixed screening intervals specifically for adults born preterm. Birth history should ideally be documented during pediatric care and again during transition to adult care. In adults, it should be asked at the first preventive cardiovascular assessment, particularly in patients with early-onset hypertension, unexplained dyspnea, reduced exercise tolerance, renal dysfunction, diabetes, or premature cardiovascular disease. For lower-risk individuals born late preterm without neonatal complications, cardiovascular reassessment can follow routine preventive-care intervals. For individuals born very or extremely preterm, those with very low birth weight, bronchopulmonary dysplasia, neonatal hypertension, neonatal kidney injury, fetal growth restriction, or persistent childhood hypertension, annual blood pressure review and periodic cardiometabolic and renal reassessment may be reasonable, with earlier reassessment if abnormal findings or symptoms develop [10,11,18,19].

In intermediate- or higher-risk patients, additional screening may include fasting lipid profile, HbA1c or fasting glucose, serum creatinine, estimated glomerular filtration rate, and urine albumin-to-creatinine ratio when clinically appropriate. This is particularly relevant because preterm birth has been associated with chronic kidney disease, smaller renal size, altered renal physiology, and diabetes [19,20]. These tests are inexpensive, widely available, and directly linked to preventive cardiology decisions.

Cardiopulmonary testing should be targeted rather than universal. Echocardiography, pulmonary function testing, exercise testing, or specialist referral may be reasonable in preterm-born individuals with unexplained dyspnea, reduced exercise tolerance, suspected pulmonary hypertension, abnormal cardiac examination, persistent hypertension, renal dysfunction, or premature cardiovascular events [23,24]. Advanced imaging should not be recommended for all individuals born preterm because current evidence does not support universal screening. The most defensible strategy is earlier recognition of modifiable risk factors and a lower threshold for evaluation when symptoms or abnormal clinical findings are present.

**Figure 1:**
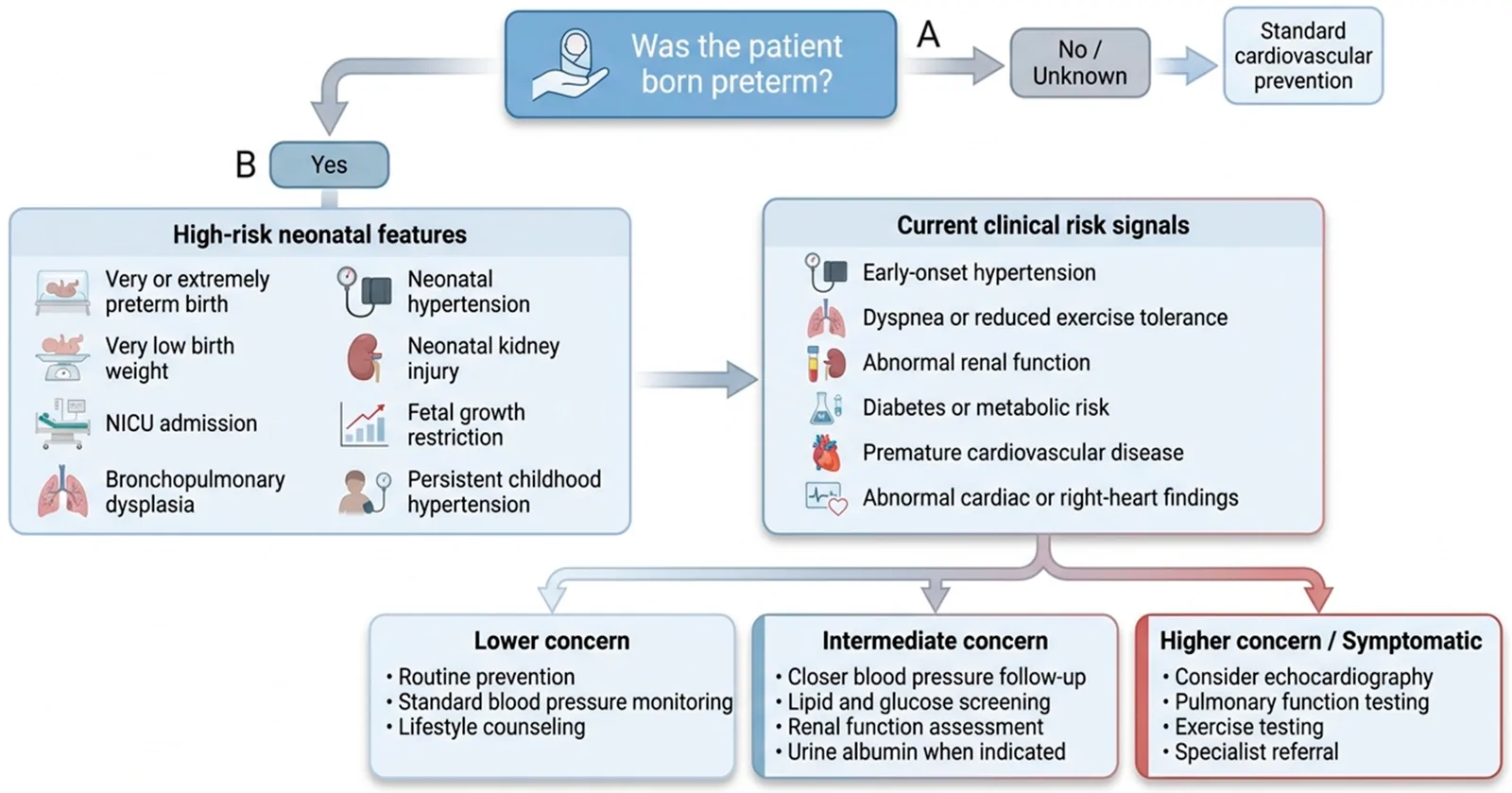
Birth-history-informed cardiovascular risk assessment algorithm. The figure summarizes a practical approach for incorporating preterm birth history into cardiovascular risk assessment. High-risk neonatal features and current clinical signals may guide blood pressure monitoring, renal and metabolic screening, symptom-guided cardiopulmonary evaluation, and targeted prevention rather than universal advanced testing. Abbreviations: NICU, neonatal intensive care unit. Created in BioRender. Alabid, I. (2026). https://BioRender.com/qa4crse (accessed on 24 July 2026).

Overall, birth-history-informed cardiovascular assessment is not an additional diagnostic burden; it is a refinement of clinical history-taking. By asking a small number of targeted questions, clinicians may identify a subgroup of patients who would benefit from earlier prevention, closer blood pressure surveillance, cardiometabolic screening, or symptom-guided cardiopulmonary evaluation. This approach translates the growing evidence on prematurity and cardiovascular risk into a practical framework for preventive care. A practical birth-history-informed risk assessment pathway is proposed in Figure 1, emphasizing risk stratification by neonatal history, current clinical findings, and symptom-guided escalation rather than universal advanced testing.

## 5. Clinical Evidence Across the Cardiovascular Spectrum

### 5.1. Hypertension and Blood Pressure Trajectories

Blood pressure is the most clinically accessible marker linking preterm birth with later cardiovascular risk. Several studies have reported higher blood pressure or altered blood pressure patterns among children, adolescents, and adults born preterm, although the magnitude of difference varies by age, gestational age, fetal growth, and neonatal course [12,36]. In childhood, extremely preterm birth has been associated with normal but slightly higher blood pressure compared with term-born peers, suggesting that vascular differences may be detectable before overt hypertension develops [21]. In adolescence, very preterm birth combined with fetal growth restriction appears to confer additional vulnerability, particularly among boys, highlighting the importance of considering sex and fetal growth when interpreting risk [10]. In adult women, Women’s Health Initiative data further linked self-reported preterm birth with prevalent hypertension, early-onset hypertension, and incident hypertension, supporting the relevance of birth history to cardiovascular risk assessment in women [37].

Adult data further support the relevance of blood pressure surveillance. Young adults born very preterm have been shown to exhibit higher blood pressure, smaller kidney size, and altered renal-related physiology, suggesting that developmental renal pathways may contribute to later hypertension [18]. Physiologic stress testing may also reveal abnormalities that are not evident at rest. Adults born preterm have demonstrated increased aortic stiffness and exaggerated blood pressure responses during exercise, supporting the concept of reduced vascular reserve under hemodynamic stress [13]. More recent pediatric evidence also indicates that preterm infants requiring neonatal intensive care, especially those with major neonatal complications, have an increased risk of persistent childhood hypertension [11]. Together, these findings make blood pressure the most practical first target for birth-history-informed cardiovascular prevention.

Among all cardiovascular signals associated with preterm birth, blood pressure is the most reproducible and clinically actionable. The available evidence does not suggest that all preterm-born individuals develop hypertension; rather, it supports a shift in blood pressure distribution, with higher risk in those born very or extremely preterm, those with fetal growth restriction, and those exposed to neonatal complications [10,11]. This makes repeated and accurately confirmed blood pressure measurement more appropriate than one-time screening alone. When office blood pressure is elevated, confirmation with repeat office or out-of-office measurements should follow contemporary hypertension guidance [35].

### 5.2. Heart Failure and Ischemic Heart Disease

The association between preterm birth and heart failure is one of the strongest arguments for considering prematurity in cardiovascular history-taking. A large Swedish cohort found that preterm birth was associated with increased risk of heart failure from childhood into adulthood, with higher risks among those born at earlier gestational ages [6]. Earlier register-based evidence also demonstrated a strong association between birth before 32 weeks of gestation and heart failure in childhood and young adulthood, although absolute event rates at young ages remained low [38]. These findings suggest that prematurity may contribute to cardiac vulnerability long before the age at which conventional heart failure risk prediction is usually applied. These heart failure findings are important because they are supported by large registry-based cohorts and show stronger associations at lower gestational ages [6,38]. However, the clinical interpretation should remain proportional: heart failure remains uncommon in early adulthood, and preterm birth should be viewed as a risk-enhancing exposure rather than as a direct predictor of disease in an individual patient.

Preterm birth has also been linked with ischemic heart disease in adulthood. In a national cohort, preterm and early-term birth were associated with higher ischemic heart disease risk, supporting the possibility that gestational age contributes to premature atherosclerotic or ischemic risk [7]. This does not imply that prematurity alone determines ischemic heart disease; rather, it may lower the threshold at which adult risk factors such as hypertension, diabetes, dyslipidemia, smoking, and obesity produce clinical disease. Therefore, a history of preterm birth may be particularly relevant when evaluating younger adults with early ischemic symptoms or multiple borderline risk factors. The ischemic heart disease association is also best interpreted within a cumulative-risk framework. Preterm birth may increase vulnerability, but adult risk factors such as hypertension, diabetes, dyslipidemia, obesity, smoking, and physical inactivity likely determine whether this vulnerability becomes clinically expressed [7].

### 5.3. Stroke and Atrial Fibrillation

The cardiovascular implications of preterm birth extend beyond heart failure and ischemic disease. A national cohort and cosibling study reported that adults born preterm had increased risks of both hemorrhagic and ischemic stroke, with stronger associations among those born earlier in gestation [9]. This supports the idea that prematurity may influence cerebrovascular risk through several pathways, including blood pressure, vascular structure, renal development, and metabolic factors. Mendelian randomization evidence has also suggested a potential causal association between early preterm birth and certain stroke subtypes, with impaired pulmonary function identified as a possible mediator [39]. Although genetic instrumental-variable studies require cautious interpretation, they add mechanistic plausibility to observational findings.

Atrial fibrillation is another relevant outcome because it links prematurity with rhythm vulnerability and atrial remodeling. A multinational cohort study found that preterm birth was associated with increased atrial fibrillation risk up to middle age, independent of measured familial factors [8]. This finding is consistent with imaging evidence showing differences in atrial structure and function among adults born preterm [15]. Although atrial fibrillation remains uncommon at younger ages, its association with prematurity strengthens the argument that birth history may be relevant across multiple cardiovascular phenotypes rather than only hypertension.

### 5.4. Renal and Cardiometabolic Outcomes Relevant to Cardiovascular Risk

Renal and metabolic disease are not purely cardiovascular outcomes, but they are central to cardiovascular prevention. The renal pathway is particularly relevant because nephrogenesis continues until late gestation, making very preterm birth biologically plausible as a contributor to reduced nephron endowment, smaller renal size, and later blood pressure vulnerability [18]. Population-level evidence has linked preterm birth with increased risk of chronic kidney disease from childhood into mid-adulthood, with stronger associations at earlier ages and lower gestational ages [19]. Therefore, renal function and albuminuria assessment may be particularly relevant in selected preterm-born individuals with hypertension, very low birth weight, neonatal kidney injury, or persistent cardiometabolic abnormalities.

Cardiometabolic risk should be considered as a complementary pathway. Preterm birth has been associated with increased risks of type 1 and type 2 diabetes from childhood into adulthood [20]. Adult cohort data also suggest that higher early-life medical risk among preterm-born individuals may be associated with higher systolic blood pressure, less favorable lipid markers, and altered body composition [40]. These findings support a practical prevention strategy focused on blood pressure, weight, glucose, lipid profile, physical activity, smoking or vaping exposure, and lifestyle counseling rather than routine advanced cardiac imaging for all individuals born preterm.

### 5.5. Composite Cardiovascular Disease and Overall Interpretation

The broadest evidence comes from large register-based studies evaluating cardiovascular disease in early adulthood. A Swedish–Danish cohort found that both preterm birth and small-for-gestational-age status were associated with increased cardiovascular disease risk, with sibling comparisons suggesting that the association with gestational age was less explained by shared familial factors than the association with fetal growth [27]. This distinction is important because it supports prematurity as an exposure with independent clinical relevance, while also acknowledging that fetal growth, maternal health, genetics, and shared environment may modify risk.

Overall, the most meaningful and clinically actionable phenotype after preterm birth is higher blood pressure across childhood, adolescence, and adulthood. Additional well-supported domains include altered cardiac structure or reserve, heart failure, ischemic heart disease, chronic kidney disease, and diabetes. Stroke and atrial fibrillation are supported by large cohort evidence but should be interpreted as later cardiovascular outcomes rather than routine screening targets in all preterm-born individuals. Pulmonary vascular and right-heart abnormalities are most relevant in individuals born very or extremely preterm, those with bronchopulmonary dysplasia, or those presenting with dyspnea, exercise limitation, or suspected pulmonary hypertension. The clinical implication is not that every individual born preterm requires intensive cardiovascular testing. Rather, preterm birth should be recognized as a risk-enhancing history item that may justify earlier surveillance, stricter attention to modifiable risk factors, and targeted evaluation when symptoms or additional risk factors are present.

The most representative studies supporting the clinical and mechanistic framing of preterm birth as an early-life cardiovascular risk enhancer are summarized in Table 2. These studies were selected to reflect the main evidence domains discussed in this review, including heart failure and ischemic heart disease [6,7], stroke and atrial fibrillation [8,9], chronic kidney disease and diabetes [19,20], and left ventricular remodeling, pulmonary vascular disease, childhood hypertension, and early-adult cardiovascular disease risk [14,23,27].

To complement the evidence-domain summary in Table 2, Table 3 provides representative effect estimates to illustrate the approximate magnitude of selected cardiovascular, renal, and metabolic associations after preterm birth.

## 6. Mechanistic Pathways and the Preterm Cardiovascular Phenotype

### 6.1. Cardiac Remodeling and Reduced Cardiovascular Reserve

Preterm birth may interrupt late-gestation myocardial maturation and expose the immature heart to postnatal circulation before normal fetal cardiac growth is complete. Cardiovascular magnetic resonance studies have shown that adults born preterm may have distinct left ventricular geometry, altered ventricular mass, and differences in systolic and diastolic functional parameters compared with term-born controls [14,42]. Right-sided remodeling is also important. Young adults born preterm have demonstrated smaller right ventricular volumes, higher right ventricular mass, and reduced right ventricular systolic function, suggesting that prematurity affects both ventricles rather than only the systemic circulation [43]. Early postnatal factors may also contribute to later cardiac dimensions, as neonatal intakes and hyperglycemia have been associated with left-heart and aortic dimensions in children born extremely preterm [44].

More recent studies support the concept of a compensated cardiac phenotype. Adolescents and adults born preterm may show smaller biventricular chamber size or altered cardiac dimensions while maintaining apparently preserved resting ejection fraction [16,45]. Similarly, school-aged children with a history of preterm birth may show altered systolic and diastolic indices on advanced echocardiography despite similar conventional measurements [46]. This distinction is clinically relevant because normal resting systolic function does not necessarily indicate normal cardiovascular reserve. Under physiologic stress, including exercise or hypoxia, individuals born preterm may demonstrate abnormal contractile responses, reduced stroke volume augmentation, or altered right-heart flow dynamics [17,25,26]. Therefore, the preterm heart may appear stable at rest but become vulnerable when exposed to increased hemodynamic demand.

### 6.2. Vascular Stiffness and Blood Pressure Physiology

Systemic vascular changes provide a major link between preterm birth and later cardiovascular disease. Individuals born preterm may have higher blood pressure, increased aortic stiffness, and abnormal vascular responses during exercise [13]. These findings may reflect impaired elastin development, endothelial dysfunction, oxidative stress, and vascular remodeling during a critical period of arterial maturation [47]. Even modest increases in vascular stiffness may become clinically relevant over time because they increase cardiac afterload and may contribute to hypertension, ventricular remodeling, and premature cardiovascular disease.

Blood pressure differences after preterm birth may be subtle in early childhood but more evident later or during physiologic challenge. Children born extremely preterm may have normal but slightly higher blood pressure than term-born peers, while adolescents born very preterm with fetal growth restriction may show sex-specific vulnerability [10,21]. This suggests that vascular risk develops along a trajectory influenced by gestational age, fetal growth, sex, renal development, and postnatal exposures, rather than appearing as a single uniform abnormality. These vascular changes should not be assumed to affect all vascular beds uniformly. Most available clinical evidence in preterm-born individuals relates to systemic blood pressure, aortic stiffness, endothelial function, or exercise hemodynamics, whereas direct evidence on coronary vascular function is more limited. It is therefore more appropriate to interpret preterm-associated vascular dysfunction as a heterogeneous vascular phenotype that may influence central arterial stiffness, peripheral vascular regulation, and possibly coronary microvascular reserve, rather than as a uniform abnormality across all arterial territories [13,47]. Future studies evaluating coronary endothelial function, coronary flow reserve, and microvascular dysfunction may help clarify whether preterm birth contributes directly to premature coronary vascular susceptibility.

### 6.3. Pulmonary Vascular and Right-Heart Pathway

The pulmonary vascular pathway is particularly important because lung and pulmonary vascular development are incomplete at the time of very preterm birth. Bronchopulmonary dysplasia, oxygen exposure, mechanical ventilation, and impaired alveolar-capillary development may contribute to pulmonary vascular remodeling and later right-heart stress [23]. Young adults born preterm have shown evidence of early pulmonary vascular disease, including abnormal pulmonary vascular function, even without overt adult cardiopulmonary disease [23]. Multimodality imaging has also demonstrated reduced right ventricular function in moderately preterm-born adults, supporting a right-heart phenotype that may not be fully explained by pulmonary physiology alone [48]. Longitudinal data in survivors of extreme prematurity also show that respiratory and cardiovascular differences can persist into late adolescence [28].

Impaired right ventricular–pulmonary arterial coupling provides a plausible mechanism for exertional symptoms and reduced reserve in selected survivors of prematurity [24]. Dynamic contrast-enhanced magnetic resonance imaging has also demonstrated pulmonary microvascular abnormalities in adult survivors of prematurity, supporting the concept that pulmonary vascular changes can persist beyond infancy [49]. Clinically, this pathway is important because unexplained dyspnea, exercise limitation, or suspected pulmonary hypertension in a person born preterm should prompt consideration of a cardiopulmonary developmental phenotype rather than being attributed only to deconditioning or obesity. Abnormal pulmonary vascular function may also provide a pathway toward pulmonary hypertension and secondary right-heart dysfunction in susceptible individuals, particularly those born very or extremely preterm or those with bronchopulmonary dysplasia. However, the current evidence does not support assuming that all preterm-born individuals develop clinically significant pulmonary hypertension. Rather, pulmonary vascular abnormalities should be interpreted as a risk marker that may become clinically relevant when accompanied by dyspnea, reduced exercise tolerance, abnormal right-heart findings, or a history of severe neonatal lung disease [23,24,30,48].

### 6.4. Renal-Developmental Pathway

Renal development is a key mechanistic pathway linking preterm birth with later cardiovascular risk because nephrogenesis continues until late gestation. Birth before completion of nephrogenesis may reduce nephron endowment, leading to compensatory glomerular hyperfiltration, smaller kidney size, increased susceptibility to albuminuria, and higher blood pressure later in life [18,32]. This pathway is clinically relevant because reduced nephron number can increase single-nephron workload and may contribute over time to hypertension, renal injury, and vascular disease.

Human studies support this developmental renal model. In young adults born very preterm, smaller kidney size, higher urinary albumin-to-creatinine ratio, and higher ambulatory blood pressure have been reported compared with term-born controls, despite similar estimated glomerular filtration rate [18]. In adolescents born extremely preterm, renal follow-up studies have also reported markers of early kidney vulnerability, including albuminuria, elevated blood pressure, or reduced kidney volumes [50]. These findings suggest that renal vulnerability may be detectable before clinically apparent chronic kidney disease develops.

Population-level evidence further supports the renal pathway. Preterm birth has been associated with increased risk of chronic kidney disease from childhood into mid-adulthood, with stronger associations at earlier ages and lower gestational ages [19]. Neonatal kidney injury, nephrotoxic medication exposure, sepsis, prolonged intensive care, and rapid postnatal growth may further modify this risk, although the relative contribution of each factor remains incompletely defined [32].

Clinically, these data do not justify universal renal testing for all individuals born preterm. However, serum creatinine, estimated glomerular filtration rate, and urine albumin-to-creatinine ratio may be reasonable in selected patients with very or extremely preterm birth, very low birth weight, neonatal kidney injury, hypertension, diabetes, or persistent cardiometabolic risk. This approach links renal surveillance to measurable clinical risk rather than prematurity alone.

### 6.5. Metabolic and Autonomic Pathways

Metabolic dysregulation may further amplify cardiovascular vulnerability after preterm birth. Large cohort evidence has linked preterm birth with increased risks of both type 1 and type 2 diabetes, with associations persisting into adulthood [20]. Adult follow-up data also suggest that higher early-life medical risk among individuals born preterm may be associated with adverse cardiometabolic markers, including higher systolic blood pressure, less favorable lipid markers, and altered body composition [40]. In addition, lower physical activity in some children born extremely preterm may contribute to reduced fitness and long-term cardiometabolic risk [51]. Dynamic FDG PET imaging suggests that cardiac metabolic remodeling may also be measurable in adults born preterm, although its clinical implications require further study [52].

Autonomic regulation is an emerging pathway. Adults born preterm with very low birth weight have been reported to show higher heart rate and blood pressure, with evidence of altered heart rate variability in mid-adulthood [22]. Reduced parasympathetic activity and sympathetic predominance may contribute to higher blood pressure, impaired vascular regulation, and reduced adaptability to physiologic stress. Although more evidence is needed, autonomic dysfunction provides a biologically plausible link between early developmental stress and later cardiovascular regulation.

### 6.6. Integrated Preterm Cardiovascular Phenotype

The preterm cardiovascular phenotype is best understood as a multisystem developmental phenotype rather than a single disease pathway. The proposed preterm heart–brain axis further illustrates how cardiovascular alterations after preterm birth may interact with other developmental systems and modifiable risk factors [53]. Interrupted cardiac growth, systemic vascular stiffness, pulmonary vascular remodeling, reduced renal reserve, metabolic dysregulation, and autonomic imbalance may interact across the lifespan [5,54]. These abnormalities may remain subclinical for years, especially when resting measures are normal, but may become clinically relevant during growth, exercise, hypoxia, pregnancy, cardiometabolic stress, or aging. This mechanistic framework supports the clinical argument that preterm birth should be documented and interpreted as part of cardiovascular risk assessment, particularly when additional risk factors or early symptoms are present.

## 7. Clinical Implications and Practice Points

The main clinical implication is that preterm birth should be treated as part of cardiovascular history rather than as an isolated neonatal detail. In pediatric and transition-of-care settings, gestational age, birth weight, neonatal intensive care admission, bronchopulmonary dysplasia, neonatal hypertension, neonatal kidney injury, and major neonatal complications should be documented before patients enter adult care [3,5]. In adult practice, this information may help contextualize early-onset hypertension and renal vulnerability [11,19], as well as unexplained dyspnea or reduced exercise tolerance in selected preterm-born individuals [23,25,26].

For primary care clinicians, blood pressure surveillance is the most practical starting point. Individuals born very or extremely preterm, particularly those with fetal growth restriction or neonatal complications, may benefit from closer blood pressure tracking during adolescence and young adulthood [10,11]. Blood pressure assessment should be interpreted using standard hypertension guidance, while recognizing that preterm birth may modify the clinical meaning of early or borderline abnormalities [35]. Routine prevention should also address body mass index, physical activity, smoking or vaping exposure, sleep, diet, family history, glucose status, and lipid profile.

For cardiologists, prematurity may be relevant when evaluating young patients with heart failure or ischemic heart disease [6,7], as well as atrial fibrillation or stroke [8,9]. Birth history may also help contextualize imaging findings such as atrial remodeling, smaller cardiac dimensions, altered right ventricular structure, or preserved resting function with reduced physiologic reserve [15,16]. Right-ventricular changes and reduced reserve may be particularly relevant in patients born extremely preterm or those with bronchopulmonary dysplasia [30,43].

Cardiopulmonary symptoms deserve targeted attention rather than universal testing. Pulmonary vascular disease and impaired right ventricular–vascular coupling have been described in selected preterm-born individuals [23,24], while exercise-based and pulmonary microvascular studies provide additional support for symptom-guided evaluation [25,26,49]. Therefore, echocardiography, pulmonary function testing, exercise testing, or specialist referral should be considered when symptoms or abnormal findings justify further assessment.

Renal and metabolic prevention should be incorporated into the same framework. Assessment of renal function, urine albumin, fasting glucose or HbA1c, and lipid profile may be particularly relevant in patients with very low birth weight, neonatal kidney injury, hypertension, diabetes, obesity, or other cardiometabolic abnormalities [18,19,20].

The message to patients should remain balanced. Preterm birth is not destiny, and many individuals born preterm remain healthy. The practical aim is earlier recognition of preventable risk, not overmedicalization. A concise clinical approach is to ask about birth history, identify high-risk neonatal features, monitor blood pressure and cardiometabolic risk, and escalate evaluation only when symptoms or abnormal findings justify it.

## 8. Limitations of Current Evidence

Several limitations should be considered when interpreting the current evidence. First, much of the literature linking preterm birth with later cardiovascular outcomes is observational. Large registry studies, sibling-comparison designs, and Mendelian randomization analyses strengthen causal inference, but residual confounding from maternal health, genetics, fetal growth, socioeconomic status, childhood environment, and adult lifestyle cannot be fully excluded [9,27,39]. Therefore, preterm birth should be interpreted as a cardiovascular risk-enhancing exposure rather than a deterministic cause of disease.

Second, preterm birth is a heterogeneous exposure. Individuals born late preterm without major complications are unlikely to have the same risk profile as those born extremely preterm with bronchopulmonary dysplasia, neonatal hypertension, neonatal kidney injury, fetal growth restriction, or prolonged neonatal intensive care admission [11,29]. This heterogeneity limits the ability to apply uniform screening recommendations to all preterm-born individuals.

Third, many cardiovascular imaging and physiologic studies include relatively small cohorts. These studies provide important mechanistic insight into ventricular remodeling, pulmonary vascular function, exercise response, and autonomic regulation, but their findings may not be generalizable to all preterm-born populations [17,22]. Differences in imaging methods, participant age, neonatal era, and definitions of prematurity further complicate comparison across studies.

Fourth, evidence remains uneven across the lifespan. Childhood, adolescence, young adulthood, and mid-adulthood are increasingly studied, but data in older adults born preterm remain limited. This matters because major cardiovascular outcomes, including heart failure and ischemic heart disease [6,7], as well as stroke and atrial fibrillation [8,9], may become more apparent with aging and cumulative exposure to adult cardiovascular risk factors. Chronic kidney disease and diabetes may also become increasingly clinically relevant across later adulthood [19,20].

Finally, no universally accepted guideline currently defines cardiovascular screening thresholds for adults born preterm. Current evidence supports routine birth-history assessment and targeted prevention, but not universal advanced imaging or intensive testing for all individuals born preterm [5]. This distinction is essential to recognize clinically meaningful risk without overmedicalizing prematurity.

## 9. Future Directions

Future research should move from documenting associations toward defining how birth history can be used in cardiovascular prevention. First, longer follow-up into later adulthood is needed. Many current studies focus on childhood, adolescence, young adulthood, or mid-adulthood, while the full burden of heart failure and ischemic heart disease [6,7], as well as stroke and atrial fibrillation [8,9], may become more clinically apparent with aging. The long-term burden of chronic kidney disease and diabetes may likewise become clearer with advancing age [19,20].

Second, future cohorts should improve risk stratification by gestational age, birth weight, fetal growth, sex, neonatal intensive care admission, bronchopulmonary dysplasia, neonatal hypertension, neonatal kidney injury, and major neonatal complications. Current evidence suggests that cardiovascular risk is heterogeneous rather than uniform across all individuals born preterm [10,11]. More precise phenotyping would help identify which patients need closer follow-up while avoiding unnecessary overmedicalization of lower-risk individuals.

Third, studies should determine which screening tools provide the greatest clinical value. Blood pressure monitoring is the most practical and immediately actionable approach, but the role of ambulatory blood pressure monitoring, renal markers, echocardiography, pulmonary function testing, cardiopulmonary exercise testing, and advanced cardiovascular imaging remains insufficiently defined [13,18]. Future studies should evaluate whether targeted screening improves clinical outcomes, not only whether it detects subclinical abnormalities.

Fourth, birth history should be tested in cardiovascular risk prediction models. Existing adult risk tools generally do not include gestational age, birth weight, or neonatal complications, which may limit risk estimation in young adults with premature or borderline cardiovascular risk [7,27]. Incorporating early-life variables could improve prevention strategies, particularly for patients who appear low risk by traditional adult risk scores but have high-risk birth histories.

Fifth, intervention studies are needed to determine whether earlier lifestyle counseling, structured exercise, blood pressure control, smoking and vaping prevention, weight management, renal surveillance, or metabolic screening can reduce long-term cardiovascular risk in people born preterm [20,51]. Finally, future research should include more diverse ethnic, socioeconomic, and healthcare settings, as much of the current evidence comes from high-income cohorts. Broader representation is essential before birth-history-informed cardiovascular prevention can be translated into widely applicable clinical guidance.

## 10. Conclusions

Preterm birth should be recognized as more than a neonatal event; it represents an early-life exposure with potential cardiovascular relevance across the lifespan. Current evidence supports an association between prematurity and a broad cardiovascular phenotype, including elevated blood pressure, altered cardiac structure and reserve, pulmonary vascular vulnerability, renal and metabolic risk, autonomic dysregulation, and increased risk of major cardiovascular outcomes. These findings suggest that birth history may help identify individuals whose risk is underestimated by conventional adult cardiovascular assessment.

The clinical value of this evidence lies in targeted prevention rather than universal testing. Gestational age, birth weight, neonatal intensive care admission, bronchopulmonary dysplasia, neonatal hypertension, neonatal kidney injury, and major neonatal complications should be incorporated into cardiovascular history-taking, particularly in patients with early-onset hypertension, unexplained dyspnea, reduced exercise tolerance, renal dysfunction, diabetes, or premature cardiovascular disease. Blood pressure monitoring, cardiometabolic screening, renal assessment when indicated, and symptom-guided cardiopulmonary evaluation offer a practical and proportionate approach.

Reframing birth history as cardiovascular history provides an opportunity to shift care from late recognition of disease toward earlier risk identification, individualized prevention, and more informed cardiovascular follow-up for the growing population of individuals born preterm.

## Figures and Tables

**Table 1:** Suggested birth-history questions for cardiovascular assessment.

Domain	Suggested Question	Clinical Relevance
Prematurity status	Were you born early or premature?	Identifies preterm birth as a potential early-life cardiovascular risk enhancer [3,5].
Gestational age	Do you know how many weeks of pregnancy you were born at? Were you born very preterm, defined as birth before 32 completed weeks, or extremely preterm, defined as birth before 28 completed weeks?	Helps distinguish late, moderate, very, and extremely preterm birth, which may carry different levels of long-term cardiovascular risk [6,7,27].
Birth weight/fetal growth	Do you know your birth weight? Were you low birth weight, defined as less than 2500 g, very low birth weight, defined as less than 1500 g, or small for gestational age/growth restricted?	Low birth weight, very low birth weight, and fetal growth restriction may suggest greater developmental, renal, and cardiometabolic vulnerability [10,18,27].
Neonatal intensive care	Did you require admission to a neonatal intensive care unit?	Indicates greater neonatal illness severity and may identify patients who need closer blood-pressure or cardiometabolic follow-up [11].
Respiratory support	Did you need oxygen, ventilation, or surfactant after birth?	May suggest neonatal lung disease and later pulmonary vascular or right-heart vulnerability, especially after very or extremely preterm birth [23,28].
Bronchopulmonary dysplasia	Were you diagnosed with bronchopulmonary dysplasia or chronic lung disease of prematurity?	Relevant to dyspnea, exercise limitation, pulmonary vascular disease, and right-heart stress in selected preterm-born individuals [23,29,30].
Neonatal complications	Did you have neonatal hypertension, kidney injury, sepsis, or other major neonatal complications?	May identify patients who need closer blood-pressure, renal, or cardiometabolic surveillance [11,31,32].
Maternal pregnancy history	Did your mother have preeclampsia, hypertension, or diabetes during pregnancy?	Provides context for shared vascular, metabolic, and familial risk, especially when interpreting pregnancy-related vascular history [33].

This table summarizes practical birth-history items that may help identify preterm-born individuals who could benefit from closer cardiovascular, renal, metabolic, or cardiopulmonary surveillance. Standard definitions are included to improve clinical clarity for adult-care settings. References are provided within the clinical relevance column to support each listed item.

**Table 2:** Characteristics of representative studies included in the review.

Study	Study Design/Population Focus	Main Domain	Key Finding	Relevance to This Review
Crump et al., 2021 [6]	Population-based cohort	Heart failure	Preterm birth was associated with increased long-term risk of heart failure into adulthood.	Supports prematurity as a clinically relevant risk marker for later heart failure.
Crump et al., 2019 [7]	National cohort	Ischemic heart disease	Preterm and early-term birth were associated with increased ischemic heart disease risk in adulthood.	Links gestational age with premature ischemic cardiovascular risk.
Crump et al., 2021 [9]	National cohort and cosibling analysis	Stroke	Adults born preterm had increased risks of ischemic and hemorrhagic stroke.	Extends the preterm cardiovascular phenotype to cerebrovascular disease.
Yang et al., 2023 [8]	Multinational cohort	Atrial fibrillation	Preterm birth was associated with increased atrial fibrillation risk up to middle age.	Supports the relevance of rhythm vulnerability and atrial remodeling.
Makker et al., 2025 [11]	Pediatric cohort	Childhood hypertension	Prematurity with neonatal complications was associated with persistent childhood hypertension.	Identifies blood pressure as a practical early surveillance target.
Crump et al., 2019 [19]	National cohort	Chronic kidney disease	Preterm birth was associated with increased chronic kidney disease risk from childhood into mid-adulthood.	Supports a renal-developmental pathway linking prematurity with cardiovascular risk.
Crump et al., 2020 [20]	National cohort	Diabetes and cardiometabolic risk	Preterm birth was associated with higher risks of type 1 and type 2 diabetes.	Supports cardiometabolic screening in selected preterm-born individuals.
Lewandowski et al., 2013 [14]	Cardiovascular magnetic resonance study	Cardiac remodeling	Adults born preterm showed differences in left ventricular mass, geometry, and function.	Demonstrates structural cardiac remodeling after preterm birth.
Goss et al., 2018 [23]	Physiologic and imaging study	Pulmonary vascular disease	Young adults born preterm showed evidence of early pulmonary vascular disease.	Supports pulmonary vascular and right-heart vulnerability.
Lu et al., 2023 [27]	Swedish–Danish cohort with familial-factor analysis	Composite cardiovascular disease	Gestational age and birth weight were associated with cardiovascular disease risk in early adulthood.	Provides broad population-level support for birth-history-informed cardiovascular risk assessment.

This table summarizes ten key studies selected to represent the main epidemiological, clinical, imaging, renal, metabolic, and pulmonary vascular evidence linking preterm birth with cardiovascular risk across the lifespan.

**Table 3:** Approximate magnitude of selected cardiovascular, renal, and metabolic associations after preterm birth.

Domain	Exposure/Comparison	Outcome	Reported Magnitude	Clinical Interpretation
Blood pressure	VLBW, defined as birth weight < 1500 g, compared with term or normal-birth-weight controls	Adult blood pressure	SBP +3.4 mmHg; DBP +2.1 mmHg	Blood-pressure elevation is modest at the individual level but clinically important at the population level and supports BP surveillance in adults born VLBW [41].
Childhood hypertension	Preterm birth with NICU admission and major neonatal complication compared with full-term birth without NICU admission or major complication	Persistent childhood hypertension	Adjusted RR 1.87; adjusted HR 2.37	Neonatal complexity identifies a higher-risk subgroup for childhood and transition-age BP surveillance [11].
Heart failure	Preterm birth compared with full-term birth	Heart failure from childhood into adulthood	Adult-age adjusted HR 1.42; extremely preterm adjusted HR 4.72	Relative risk is higher after severe prematurity, but absolute event rates in young adulthood remain low [6].
Ischemic heart disease	Preterm birth compared with full-term birth	Ischemic heart disease in adulthood	Adjusted HR 1.53	Supports preterm birth as a risk-enhancing history item when evaluating premature ischemic risk [7].
Stroke	Preterm birth compared with full-term birth	Stroke at ages 18–43 years	Adjusted HR 1.26; early preterm adjusted HR 1.42	Association is modest overall but stronger with earlier gestational age; this supports prevention rather than routine stroke screening [9].
Atrial fibrillation	Preterm birth compared with term birth	Atrial fibrillation up to middle age	Adjusted HR 1.30; sibling-analysis HR 1.29	Suggests rhythm vulnerability after preterm birth, although atrial fibrillation remains uncommon at younger ages [8].
Chronic kidney disease	Preterm birth compared with full-term birth	Chronic kidney disease from childhood into mid-adulthood	Adjusted HR 1.94; extremely preterm adjusted HR 3.01	Renal risk is one of the stronger long-term associations and supports selective renal surveillance in higher-risk survivors [19].
Diabetes	Preterm birth compared with full-term birth	Type 1 and type 2 diabetes at ages 18–43 years	Type 1 diabetes adjusted HR 1.24; type 2 diabetes adjusted HR 1.49; extremely preterm type 2 diabetes adjusted HR 2.55	Supports cardiometabolic screening, especially in adults born very or extremely preterm or with additional cardiometabolic risk factors [20].

This table summarizes representative effect estimates to provide clinical context for the relative magnitude of associations discussed in the review. Estimates differ by study population, age at assessment, exposure definition, outcome definition, and covariate adjustment; therefore, they should be interpreted as approximate indicators of magnitude rather than directly comparable pooled effects. Abbreviations: BP, blood pressure; DBP, diastolic blood pressure; HR, hazard ratio; NICU, neonatal intensive care unit; RR, relative risk; SBP, systolic blood pressure; VLBW, very low birth weight.

## Data Availability

No new data were created or analyzed in this study. Data sharing is not applicable to this article.

## Abbreviations

The following abbreviations are used in this manuscript:

Abbreviation	Full term
AF	Atrial fibrillation
BP	Blood pressure
BPD	Bronchopulmonary dysplasia
CKD	Chronic kidney disease
CMR	Cardiovascular magnetic resonance
CVD	Cardiovascular disease
ESC	European Society of Cardiology
FDG PET	Fluorodeoxyglucose positron emission tomography
GA	Gestational age
HbA1c	Hemoglobin A1c
HF	Heart failure
IHD	Ischemic heart disease
LV	Left ventricle/left ventricular
NICU	Neonatal intensive care unit
RV	Right ventricle/right ventricular
SGA	Small for gestational age
VLBW	Very low birth weight
